# The Nervous Phenomena Observed in a Case of Exposure of the Anterior Column of the Cord from Syphlitic Ulceration of Upper Pharynx

**Published:** 1887-12

**Authors:** A. G. Hobbs

**Affiliations:** Prof. of Eye, Ear and Throat Diseases in the Southern Medical College, Atlanta, Ga.


### Southern Medical Record.

A MONTHLY JOURNAL OF PRACTICAL MEDICINE.

☞ All Communications must be addressed to R. C. WORD, M. D., Managing Editor.

"*Quicquid Præcipies Esto Brevis.*"

VOL. XVII. ATLANTA, GA., DECEMBER, 1887. No. 12.

### ORIGINAL AND SELECTED ARTICLES.

### THE NERVOUS PHENOMENA OBSERVED IN A CASE OF EXPOSURE OF THE ANTERIOR COLUMN OF THE CORD FROM SYPHILITIC ULCERATION OF UPPER PHARYNX.

BY A. G. HOBBS, M. D.,
Prof. of Eye, Ear and Throat Diseases in the Southern Medical College, Atlanta, Ga.

[Read before the American Rhinological Association and before the Section on Laryngology of the Ninth International Medical Congress.]

In October of last year, a young man was brought to my office to be treated for "ulcerated sore throat," as his father expressed it. He had been a cowboy in Texas for two years, strong and robust, weighing 140 pounds. When he reached me he was thin, pale, and anæmic, weighing only 104 pounds. He denied ever having had syphilis, nor had he any evidence of this disease, other than the ulcer about to be described, and, according to his own history, there never had been any other symptoms of syphilis.

A posterior rhinoscopic examination revealed an ugly sloughing ulcer in the posterior and superior wall of the pharynx about the size of a silver quarter. By the use of a soft palate retractor a direct view of the greater part of the ulcer could be obtained. A bent probe discovered necrosed and detached bone at a depth of about half an inch, a piece of which I succeeded in extracting at the first sitting. At each succeeding daily visit for two weeks small pieces of bone, from the size of a pin head to a small pea, were either washed out with the cleansing syringe or extracted with the probe or scoop. My index finger would now enter the cavity as far as the first joint, by which means I could discover either detached or jagged and undetached bones.

The treatment had, from the beginning, been based upon the assumption that it was a syphilitic ulcer, notwithstanding his repeated denials of ever having contracted the disease, and his father's assurances that he had been healthy from infancy. and could not, therefore, have inherited the taint. He began on 40 grains of iodide of potash, three times a day, in a menstruum of Succus Alterans. This dose was afterwards doubled. The ulcer was daily cleansed with cotton probes, syringes and sprays. Sprays were applied not only directly, but through the nose, on account of the discharge being partly expelled through that channel. Listerine was used for its disinfectant and cleansing properties, and nitrate of silver, full strength, was applied to the bottom of the cavity by a cotton probe, after which the cavity was packed with iodol.

About this time—after four or five weeks of daily treatment—the nervous phenomena first occurred. For a week or ten days, he had been complaining of constant pain in the back of his head and neck, which caused him to carry his head to one side, and rigidly. During this latter period—the fourth and fifth weeks—he had been unable to sleep, except for a few minutes at a time, and in a sitting posture. After the cavity had been thoroughly cleansed at one of the treatments, I pressed the nitrate of silver probe to its bottom, when, as suddenly as if he had been shot, one-half of the patient's body became paralyzed ; his head fell to the right, his right leg turned outward,and he would have fallen from the chair if I had not caught him. Without losing consciousness at any time, this hemiplegic condition lasted about thirty seconds, when, as he expressed it, " he felt a tingling in the right half of his body," and in another half-minute, he slowly raised his right side into position. The next day the same phenomena was repeated, *but on the opposite side.* He was now so reduced in weight (to ninety-six pounds) and in strength by the loss of sleep and constant pain, that he could not walk to my office. The extreme insomania lasted about ten days, during which time his attendants thought he did not sleep one hour in twenty-four. The pupil of the side affected became slightly dilated, but there was apparently no change in the perspiratory glands while the paralysis lasted. But from this time, healing began at the bottom of the ulcer, the discharge became less, and examinations with the finger did not discover any more rough bones ; he slept better, and complained less of the pain in the back of his head and neck.

He naturally did not desire that a paralysis should be deliberately produced now, because he was not certain, nor could I with much confidence assure him that it would only last one minute if repeated.

A week passed on with all of his symptoms gradually improving, when I could no longer resist the temptation of making another pressure with the probe. Paralysis of the side pressed upon was again exhibited, but in a much milder degree than before, ending in the same tingling sensations as described before.

As only the spray and powder blower were used from this time onward in dressing the cavity, no more pressures with the probe were made till healing had well progressed—probably ten days or two weeks after the test. To my surprise, when the probe was now pressed into the cavity a condition just the opposite of paralysis was exhibited ; the arm and leg jerked and jumped similar to a case of chorea. These choreic muscular contractions, unlike the paralysis, lasted only during the pressure of the probe. In repeating the probe pressures at intervals during the next few days, the same choreic symptoms were produced, always on the corresponding side of the pressure, but in a less and less degree, requiring a still firmer pressure to produce the effect.

Finally, when the healing process had been thoroughly established and cicatricial tissue had, in a measure, closed up the cavity, only a "tingling or prickling" sensation followed the probe pressure—a sensation similiar to that which followed the hemiplegia in the first instance. After the cicatricial tissue had thoroughly hardened, no manifestations followed the pressure of the probe. At the time of writing—ten months since I first saw the patient—he is entirely recovered, and presents a perfect picture of health. His weight is thirty to forty pounds more than he weighed in November of last year. He was kept on a lessened dose of iodide of potash and Succus Alterans until last July.

I cannot state accurately as to the total amount of dead bone taken from the cavity, but I should say that its area would about equal that of a medium sized almond.

The sequel, it would seem, proved my diagnosis to be correct, since the ulcer had gradually increased in size and virulency for six months prior to the beginning of the anti-syphilitic treatment, and though he did not improve at once, healing did begin as soon as the local treatment succeeded in cleansing the ulcer of necrosed bone.

The case is of even greater interest to the Neurologist than to the Laryngologist, and had more systematic experiments been made, and closer observations been taken of the nervous phenomena, something of greater interest might have been gained. I will confess that I had not the temerity to risk many experiments at the time in the progress of the case where they would have been available.

Some important points may be noted: (1.) The necrosis must have occurred in the spinous process of the second cervical vertebra, hence the spinal cord was exposed, either between the first and second, or the second and third vertebræ. (2.) The probe pressure always produced its effect on the corresponding side of the body. (3.) The pressure that produced the paralysis must have wounded the anterior column by pressing a jagged piece of bone against the cord, and the pressure that produced the convulsions must have only irritated the anterior column, since it is well known, by experiments on the lower animals, that a wound of the anterior column produces immediate paralysis, and, on irritation, immediate convulsions.

